# Theoretically Predicting Cyberbullying Perpetration in Youth With the BGCM: Unique Challenges and Promising Research Opportunities

**DOI:** 10.3389/fpsyg.2021.708277

**Published:** 2021-09-29

**Authors:** Christopher P. Barlett, Christi Bennardi, Sullivan Williams, Talia Zlupko

**Affiliations:** Gettysburg College, Gettysburg, PA, United States

**Keywords:** cyberbully perpetrators, cyberbullying & cyber aggression, theory, online risk, cyberbullying prediction

## Abstract

The psychological variables and processes germane to cyberbullying need additional empirical attention—especially for adolescent samples. Myriad studies and meta-analytic reviews have confirmed the deleterious psychological and behavioral consequences of being cyber-victimized. We argue that one method to curtail such effects is to inform interventions aimed at reducing cyberbullying perpetration regarding the why and for whom cyberbullying is likely. This review expands on these issues and emphasizes the Barlett Gentile Cyberbullying Model (BGCM) as the only validated cyberbullying-specific theory to predict cyberbullying perpetration. Our principal thesis is that the wealth of research validating the BGCM has been with adult samples and applying the BGCM to adolescents presents both challenging and exciting research opportunities for future research and intervention development in youth.

Today's technologically savvy youth have near instantaneous Internet accessibility at their fingertips. Indeed, findings from a 2018 Pew Research Center Study showed that 45% of US youth (aged 13–17) reported being online “almost constantly,” which is a 21% increase from 2014 to 2015 (Anderson and Jiang, 2018). While such Internet use and accessibility have undoubtedly aided in the rapid speed of communication and dissemination of ideas and knowledge, some individuals decide to engage in antisocial online behaviors, such as cyberbullying. Smith et al. (2008) defined cyberbullying as an “aggressive intentional act carried out by a group or individual, using electronic forms of contact, repeatedly and over time against a victim who cannot easily defend him or herself” (p. 376). Prevalence data shows that 37% of youth worldwide reported being cyber-victimized and 24% report cyberbullying others (Microsoft, 2012). Moreover, the same 2018 Pew Research Center Study (Anderson and Jiang, 2018) revealed that 24% of US youth indicated that social media had a mostly negative effect on their peers, and, of those youth, 27% noted that bullying and rumor spreading was the main reason for such negativity. These statistics alone beget the importance of reducing the likelihood of cyber-victimization. Barlett (2017) argued that one route to preventing cyber-victimization is to understand the processes and variables that predict cyberbullying perpetration with the ultimate goal of better developing successful cyberbullying intervention programs. The purpose of the current review is to (a) discuss recent theoretical developments elucidating the underlying processes germane to cyberbullying, (b) delve into the theoretical challenges and exciting future research possibilities that youth samples offer cyberbullying theory, and (c) discuss our primary predictions concerning intervention efforts.

## Theoretically Predicting Cyberbullying

Theory is arguably the most important part of the scientific method. Parsimonious and falsifiable theory guides hypotheses to yield scientific discoveries that help scientists understand behavior. Early, atheoretical, research was paramount to understand the scope (prevalence, sex differences, grade differences, etc.) of the “cyberbullying problem,” which eventually matured to utilize existing social psychological, communication, and sociological theories to explain why and for whom cyberbullying perpetration is more likely (c.f., Barlett, 2017, 2019). For instance, Heirman and Walrave (2012) utilized theory to predict cyberbullying perpetration from cyberbullying attitudes, social norms, and perceived behavioral control through cyberbullying intentions in a sample of Belgian youth. Various social and communication-based theories have been shown to reliably predict cyberbullying perpetration, such as General Strain Theory (Paez, 2018), Routine Activities Theory (Navarro and Jasinski, 2013), General Aggression Model (Kokkinos and Antoniadou, 2019), Social-Ecological Model (Guo et al., 2021), Uses and Gratifications (Tanrikulu and Erdur-Baker, 2021), Online Disinhibition Effect (Udris, 2014), and others.

One noteworthy limitation of applying such theories to understand malicious online behavior is the inability to differentiate cyber and traditional bullying perpetration. Barlett (2019) noted the importance of being able to theoretically predict cyberbullying incrementally from traditional bullying, despite the high correlation between these two forms of bullying (*r* = 0.45; Kowalski et al., 2014). Indeed, understanding cyberbullying perpetration incrementally from traditional bullying may offer important insights into better predicting cyberbullying and may also lead to better interventions focused on decreasing cyberbullying. Notably, there is reason to expect the theoretical processes involved in cyberbullying to differ from traditional bullying processes. Although certain predictors share common variance with both types of bullying, such as callous-unemotional traits (e.g., Antoniadou et al., 2016), low empathy (e.g., Del Rey et al., 2016), narcissism (e.g., van Geel et al., 2017), and others, Vandebosch and Van Cleemput (2008) and others (Menesini and Nocentini, 2009) noted several differences between traditional and cyberbullying that necessitate attention. First, cyberbullying involves no physical contact between the bully and the victim due to the online nature of the harm. Thus, one's physical stature (height, weight, muscle mass) is likely less important in online contexts than face-to-face situations (Barlett et al., 2017b). Second, the online environment affords an online aggressor an increased perception of anonymity (Wright, 2013, 2014), which, according to online disinhibition effect (Suler, 2004), increases the likelihood of online antisocial behaviors (Udris, 2014). Currently, there is only one empirically validated theory that predicts cyberbullying perpetration incrementally from traditional bullying while exploiting these differences between both forms of bullying: the Barlett Gentile Cyberbullying Model (BGCM; Barlett and Gentile, 2012).

The basis for the BGCM is traditional aggression-based learning theories, such as the General Learning Model (GLM; Gentile et al., 2014) and General Aggression Model (GAM; Anderson and Bushman, 2002), which explicates the importance of initial behaviors predicting subsequent behaviors. The GAM was derived to offer a more comprehensive theory of aggression compared to other domain-specific aggression-focused predecessors (e.g., Script Theory, Priming, Cognitive Neo-Association Theory, Excitation Transfer Theory, and others; c.f., Anderson and Carnagey, 2004). The GAM consists of distal and proximate processes. Briefly, the proximate GAM posits that two input factors: situational (e.g., provocation) and personality (e.g., being male) either individually or interactively influence the internal state, which consists of inter-correlated aggressive thoughts, aggressive feelings, and physiological arousal. Changes to one, or any combination, of internal state variables cause higher-order attributional processes to be engaged to yield either premeditated or impulsive aggressive or non-aggressive behavior. Knowing the input factors juxtaposed with subsequent changes to the internal state and attributional processes can accurately predict the likelihood of aggression (Gentile and Bushman, 2012). GAM further posits two feedback loops. The first is that after an enacted act of aggression the victim's response feeds back into the situational input factor to continue a possible cycle of aggressive responding. The second feedback loop has the ensuing aggressive response and subsequent victim's response lead to distal GAM processes. The distal GAM posits that continued and positively reinforced learning of aggressive actions, stimuli, etc. will eventually lead to the development of one's aggressive personality through several knowledge structures: aggressive attitudes, desensitization, aggressive scripts and schemas, and aggressive biases. Finally, the proximate and distal GAM are connected as one's aggressive personality formed using distal processing is an important personality input factor in the proximate GAM.

The GLM was derived to further explicate the learning mechanisms germane to the General Aggression Model. Gentile et al. (2014) noted the many influences that learning has on GAM processing at both the proximate and distal levels. For instance, classical and discriminate learning can influence the strength and direction in the correlations between the internal state variables. Moreover, repeated learning encounters (single episodes in the GAM) and practice can reinforce, develop, and automatize the knowledge structures that help derive one's aggressive personality.

The GLM and GAM tenets regarding how single episodes of aggression act as repeated learning encounters and practice to yield behavior are the primary theoretical underpinnings of the BGCM. For instance, Gentile and Bushman (2012) showed that the best predictor for future aggression is a history of aggression and multiple longitudinal studies have shown that early cyberbullying perpetration shows significant stability over time (e.g., Sticca et al., 2013; Zhang et al.). Indeed, a meta-analysis of longitudinal studies showed that the relationship between early and later cyberbullying perpetration was positive and significant (*r* = 0.43; Marciano et al., 2020). This relationship highlights the continued learning aspect of BGCM.

According to both the GAM and GLM, internal and/or external reinforcement for antisocial behaviors shapes the likelihood of continued behavior and learning. In their seminal work, Bandura et al. (1963) found that children are more likely to harm a toy Bobo doll if they witness an adult getting praised for aggressing against the same doll earlier. Subsequent work has confirmed that positive reinforcement from peers often leads to subsequent aggression (Jung et al., 2018). Overall, reinforcement—especially positive—helps guide and develop future behavior. Specific to cyberbullying, research has shown that positive reinforcement from friends or family correlates with cyberbullying perpetration (Barlett and Gentile, 2012). Moreover, Bastiaensens et al. (2016) found that the likelihood of a bystander joining a cyberbullying attack increased when normative pressure from friends, class group members, parents, and teachers was high, further emphasizing the role that reinforcement has on cyberbullying perpetration.

**Figure 1:**
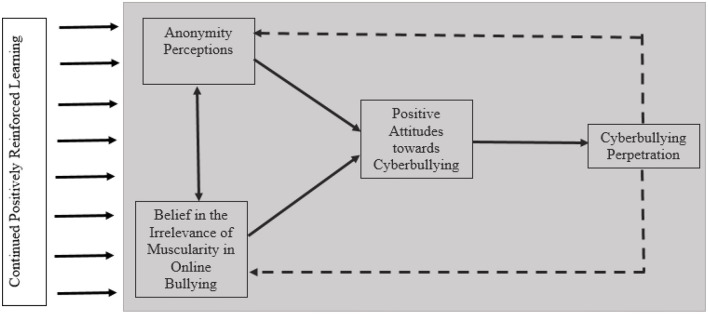
The Barlett Gentile Cyberbullying Model (BGCM).

**Figure 2:**
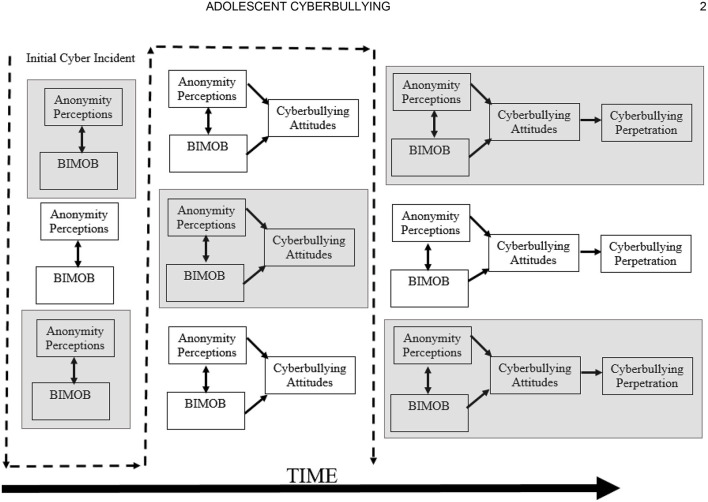
The development of the BGCM with continued experiences over time.

Figure 1 displays the current operationalization of the BGCM. Adapted from the previously noted GLM and GAM learning postulates, this model begins with the basic premise that early initial cyber-aggressive incidents act as learning trials by which the perpetrator (a) believes in the irrelevance of muscularity for online bullying (BIMOB) and (b) perceives themselves to be anonymous. As argued previously, research has suggested that BIMOB and anonymity perceptions are but two of the key differences between cyber and traditional bullying (Vandebosch and Van Cleemput, 2008). Continued positively reinforced cyber-aggressive behaviors further reinforce and automatize these, and possibly other, constructs to develop positive cyberbullying attitudes. Adhering to social psychological theory, the development of these attitudes eventually predicts cyberbullying perpetration behavior (see Barlett, 2017 for review). Figure 2 displays the temporal ordering of how cyberbullying perpetration develops in accordance with BGCM over time in conjunction with learning theory and assuming a positively reinforced online and/or in-person environment.

The tenets of the BGCM have been well-researched and validated. Indeed, scholars have shown that (a) anonymity perceptions predicts cyberbullying attitudes (Barlett, 2015), (b) BIMOB predicts cyberbullying attitudes (Barlett et al., 2017a), and (c) cyberbullying attitudes predict subsequent cyberbullying behavior (Doane et al., 2014). Moreover, longitudinal data has validated the entire model and found that Wave 2 cyberbullying attitudes mediate the relationships between (a) Wave 1 BIMOB and Wave 3 cyberbullying perpetration and (b) Wave 1 anonymity perceptions and Wave 3 cyberbullying perpetration (with waves separated by ~3 months; Barlett et al., 2017a). Moreover, the BGCM has been validated cross-culturally (Barlett et al., 2021). Finally, and most importantly, studies have shown that these effects remain while statistically controlling for traditional bullying perpetration by covarying this measured variable in primary path models (Barlett and Helmstetter, 2018).

Specifically related to the learning tenets of BGCM, Barlett and Kowalewski (2019) conducted a short-term four-wave longitudinal study (with approximately a 3 month lag between waves) with emerging adults. Scholars measured anonymity perceptions and BIMOB at Wave 1, cyberbullying attitudes at Wave 2, and cyberbullying perpetration at Wave 3. Results supported the BGCM tenets; however, and more importantly, results further showed that Wave 3 cyberbullying perpetration predicted anonymity perceptions and BIMOB measured at Wave 4. This latter finding suggests that cyberbullying behaviors continued to reinforce and increase subsequent learned cyberbullying-related knowledge structures consistent with BGCM theorizing.

Finally, empirical evidence suggests that the BGCM is robust. Indeed, the tenets of the BGCM have been shown in (a) youth (Barlett, 2015) and adults alike (Barlett and Gentile, 2012), (b) using correlational (Barlett et al., 2019) and longitudinal studies (Barlett and Kowalewski, 2019), and in several countries across the world (e.g., USA, Brazil, Australia, China, Singapore, Japan, and Germany; Barlett et al., 2021). Overall, the amount of replicated findings across multiple studies on various samples with different empirical designs suggests a valid theoretical model.

## Cyberbullying in Youth: Exciting Challenges and Future Work

We believe that the BGCM is important for understanding cyberbullying perpetration; however, one valid criticism of the BGCM is that the theoretical postulates have been largely validated on adult samples. Will the tenets of the BGCM—collectively—be substantiated in a youth sample? There are reasons to both be optimistic and pessimistic for such theoretical applications. Clearly, extensive future research is desperately needed, and, thus, we can only speculate based on existing theory and research to answer this question.

The most important issue in applying the BGCM to youth is the age of the child. First, participant age is a significant predictor of cyberbullying perpetration. Indeed, in their meta-analysis, Kowalski et al. (2014) showed a weak, yet significant, effect of age on cyberbullying perpetration (*r* = 0.05). The direction of these and other (e.g., Del Rey et al., 2016b; Barlett and Chamberlin, 2017; Beyazit et al., 2017; Cho and Yoo, 2017) effects suggest that cyberbullying increases across adolescence. The BGCM accounts for the linear relationship between age and cyberbullying via its learning postulates. In theory, younger youth may not have had many, if any at all, experiences aggressing against another person online, which effectively negates the learning tenets germane to BGCM processes. Eventually, in accordance with Figure 2, early initial antisocial online actions likely lead to the development and automatization of learned cyberbullying predictors (anonymity, BIMOB, and, eventually, cyberbullying attitudes).

There is precedent for extending the BGCM to youth samples. Indeed, portions of the BGCM have been shown valid in youth populations. For instance, Barlett (2015) used a short-term four wave longitudinal study (with time lags of ~3 months) of US adolescents (average age is 15.50 years) and showed that the relative weight of early (e.g., Wave 1 and 2) BGCM variables on predicting later (e.g., Wave 4) cyberbullying perpetration is substantial. Namely, results showed high levels of Wave 1 cyberbullying attitudes and anonymity perceptions predicted Wave 4 cyberbullying behavior. Moreover, Wright (2014) sampled US youth and showed that anonymity predicted cyberbullying perpetration (see also Wang and Ngai, 2021). Finally, extensive work has shown that cyberbullying attitudes correlate (Shim and Shin, 2016; Handono et al., 2019) with cyberbullying perpetration in youth.

However, several important, yet untested, questions remain that hinge on a new research paradigm. In order to fully test the learning postulates of the BGCM in youth, researchers must somehow reliably identify a population of children who have neither been cyber-victimized nor committed cyber-aggressive actions^1^. Then, scholars would have youth complete several questionnaires to assess the theoretical predictors of cyberbullying, such as those expounded by Kowalski et al. (2014). Using longitudinal or daily diary methods researchers would need to monitor and assess if and when youth engaged in a cyber-encounter (either sending or receiving harmful online messages) using validated measures that assess frequency of cyberbullying perpetration. Finally, scholars should continue to monitor these youth over time to assess their anonymity perceptions, BIMOB, cyberbullying attitudes, and cyberbullying perpetration. This hypothetical study can help answer key proceeding questions:

### Number of Learning Trials

The first important question that needs empirical investigation is how many learning trials are needed to initiate BGCM's learning processes? Without knowing the factors and age critical for predicting one's first cyberbullying experiences, it is difficult to theoretically predict how many learning trials are necessary to engage BGCM processes. For some, it is likely that only one cyber-aggressive action is needed to learn that they are anonymous and believe that their physical stature is irrelevant in the online world. For others, several cyber-aggressive actions are needed to achieve the same degree of learning. Although we cannot yet pinpoint the exact number of learning trials to accurately predict future cyberbullying perpetration, we can surmise that personality and learning differences likely predict the speed with which attitudes and knowledge are learned. Indeed, the General Learning Model (Gentile et al., 2014) argues that environmental (e.g., parental influences) and biological (e.g., sex) modifiers influence the extent to which people learn social behaviors. For instance, Nivette et al. (2014) found evidence to suggest that aggression was highest for males who are from countries that have high gender inequality in a sample of 7–13 year old European youth with diverse immigrant backgrounds. Moreover, recent data suggest that how “well off” a family is and residing country both differentially predict cyberbullying perpetration in a sample of youth across 41 countries (Li et al., 2020). These, and other, data suggest the influence of both environmental and biological predictors of antisocial behavior, consistent with learning theory.

In short, no published study that we are aware of has tested the number of learning trials needed to develop the knowledge structures necessary to predict cyberbullying perpetration in accordance with BGCM. However, identifying the number of cyber-aggressive trials needed likely depends on several biological and environmental modifying variables, including age, SES, country, and reinforcement.

### Predicting Cyberbullying

The second unanswered question that has theoretical bearing is: what variables predict the likelihood of one's first cyber-aggression experience? Understanding what personality and situational variables predict when youth decide to engage in their first cyber-aggressive actions has important implications for prevention. As an example, if researchers identified that owning a cellular phone predicts the first cyber-aggressive behavior, then parents and prevention experts can pair cyberbullying prevention tactics (e.g., protective factors; Kowalski et al., 2014) with cellular phone acquisition to hopefully reduce future cyberbullying. As alluded to earlier, it is likely difficult to empirically capture youth's first cyber-aggressive experience. Despite the research showing substantial mean-level changes in personality traits (e.g., agreeableness) from age 10 to 60+ (Soto et al., 2011), which may predict cyberbullying (van Geel et al., 2017), scholars could investigate personality predictors (see Kowalski et al., 2014 for several such variables) or situational predictors. A study by Englander (2018) sampled US youth (grades 3–5) and showed that the likelihood of being a cyberbully was higher if children owned a cellular phone, despite the low prevalence of cyberbullying behavior at that age (see also Englander, 2019). By extension, perhaps youth who have never cyberbullied before and are eventually provided with a cellular phone will be more likely to engage in their first cyberbullying experience than their peers who do not own a cellular phone.

## Cyberbullying Theory and Interventions

Overall, we believe that research endeavors delving into further understanding cyberbullying perpetration has both theoretical and practical implications. First, continued research into the theoretical developments should help scholars better understand the psychological mediators and moderators that predict cyberbullying perpetration. Our hope is that theory can guide such research endeavors. Many interesting research questions abound, especially as the technological landscape shifts. If future scholars choose to utilize BGCM theorizing, there are several possibilities for BGCM expansion. For instance, cyberbullying and traditional bullying differ in many ways—not just anonymity perceptions and BIMOB—that need empirical investigation. For instance, research has shown that online permanency beliefs correlate with cyberbullying perpetration (Wright, 2013) and are more important in the online than face-to-face world. Moreover, the ability for one single online aggressive act to be shared, liked, copied and pasted in other formats, and distributed to others almost instantaneously is another difference that needs empirical attention. These, and perhaps other differences, should be tested for possible integration into BGCM theorizing akin to how BIMOB and anonymity perceptions are placed. Finally, BIMOB focuses on muscularity as an estimate of power; however, other definitions of power could have theoretical implications, such as popularity.

Another possible area for future theoretical work is to examine other possible mediators that could explain why cyberbullying perpetration is likely. Recall that the BGCM explicates positive cyberbullying attitudes as the lone mediator; however, more are likely possible. Consistent with the distal GAM and GLM, cyberbullying-related scripts, schemas, and biases may also be key mediators. We are unaware of any research examining these variables, and such future work is warranted. Furthermore, cyberbullying intentions are likely a key mediator that could be integrated into BGCM. Several studies have shown that cyberbullying attitudes and behavior both correlate significantly with cyberbullying intentions (e.g., Heirman and Walrave, 2012; Pabian and Vandebosch, 2014; Auemaneekul et al., 2019).

Finally, continued work should focus on the variables that may moderate the relationships in the BGCM. We already discussed both age of participant and previous cyberbullying exposure (either as a victim or as a perpetrator); however, others likely exist. For instance, meta-analytic findings have shown that aggression, problematic Internet use, social support, and other variables predict cyberbullying perpetration (Kowalski et al., 2014). These, and other, variables could also affect BGCM processing. For example, Barlett et al. (2019) found that technology access and time spent online significantly correlated with cyberbullying attitudes and perpetration, which may suggest that various technology-related variables moderate existing BGCM relationships (such moderation tests were not conducted in the study).

In addition to the basic extensions of the BGCM, there are several applied implications that warrant consideration. Perhaps the most important is how continued validated research can further our intervention efforts to increase the efficacy of such programs. Several reviews of the literature (Espelage and Hong, 2017; Lancaster, 2018; Tanrikulu, 2018) and meta-analytic findings (Gaffney et al., 2019) have shown that cyberbullying intervention programs are mostly successful. For instance, one study had German youth (aged 11–17 years) randomly assigned to a control group, a short-term intervention group, or a long-term intervention group. For the latter two groups, participants received a Media Heroes training program—an intervention focused on teaching youth various skills (i.e., empathy), knowledge (i.e., Internet risks, legal consequences, definitions), and engaging in activities (i.e., role-playing, debates, presentations) to purportedly reduce cyberbullying (Schultze-Krumbholz et al., 2016). Results showed an (a) increase in cyberbullying for the control group over time, (b) no change in cyberbullying for those in the short-intervention group (a 1 day program with four 90 min sessions), and (c) a decrease in cyberbullying for those in the long-term intervention group (a 10 week program with one 90 min session per week; Wölfer et al., 2014).

Fortunately, several of these interventions use curricula derived directly from validated social psychological, communication, and sociological theories. For instance, Media Heroes (Chaux et al., 2016), CONRED (Del Rey et al., 2016a), and Doane et al.'s (2016) video intervention all incorporated the Theory of Planned Behavior/Reasoned Action, which posits that subjective norms, attitudes, and perceived behavioral control predict behavior indirectly through intentions. Media Heroes, for example, molded their intervention curricula onto Theory of Planned Behavior by mapping specific modules onto the tenets of the theory, such as consequences of cyberbullying onto attitudes, class climate onto subjective norms, and online self-protection onto perceived behavioral control (Wölfer et al., 2014). In our opinion, Media Heroes is a perfect example of how intervention curricula derived from theory can successfully alter cyberbullying perpetration in youth. Subsequent examples of interventions derived from other theories abound.

Despite these theoretical and practical implications, the theories used to derive intervention curricula for youth are not specific to cyberbullying. True, Media Heroes includes information about cyberbullying (e.g., legal issues); however, none of their curriculum focus on aspects of cyberbullying devoid of traditional bullying. We have already articulated the importance of such theoretical differentiation. None of the cyberbullying specific theories, such as the BGCM, are used to derive intervention theory for youth; however, such extensions are welcome. First, as already discussed, there is preliminary evidence that BGCM tenets apply to youth. Second, research has shown that an intervention that teaches individuals that they are not as anonymous as they believe can reduce anonymity perceptions, which causes changes in cyberbullying perpetration 2 months later in emerging adults (Barlett et al., 2020). Therefore, an intervention that incorporates BGCM postulates should help to reduce cyberbullying perpetration through a reduction in either anonymity perceptions, BIMOB, and/or cyberbullying attitudes. Future research should validate such intervention efforts.

One valid criticism is that intervention curricula derived from cyberbullying-specific theories are unnecessary. Indeed, if research has confirmed that interventions are already successful when cyberbullying theory is not incorporated, then why utilize cyberbullying theory? For instance, Gradinger et al. (2015) showed successful changes in cyberbullying with a more generalized anti-bullying program (ViSC) that is absent any cyberbullying instruction. This is an important and valid criticism of our argument. We are unaware of any evidence to suggest that interventions derived from cyberbullying theory are statistically different from interventions derived from other social psychological, sociological, or communication based curricula. However, perhaps an existing intervention that incorporates some module(s) or lesson(s) about issues specific to cyberbullying prediction, such as anonymity perceptions, could further enhance the success of such interventions that use traditional bullying reduction skills (e.g., empathy). This is speculation and future research should compare these interventions.

A second applied extension of our work is that parents, and other caregivers, can help reduce cyberbullying. Our central thesis is that cyberbullying is a learned behavior through the BGCM lens. Thus, parents, peers, school counselors, etc. can help reduce the likelihood of cyberbullying by disrupting the learning germane to cyberbullying. Data from several studies support such claims. For instance, poor communication with parents has been shown to positively predict cyberbullying (e.g., Romero-Abrio et al., 2019), whereas a positive communicative relationship with parents can decrease cyberbullying (e.g., Park et al., 2014).

## Final Remarks

In conclusion, we aim to present a review of the literature that we feel can add to our existing knowledge of theoretically predicting cyberbullying perpetration in youth. Since the majority of research on cyberbullying theory is validated on adults, but cyberbullying perpetration interventions are largely delivered to adolescents, there is a disconnect that needs to be addressed. We hope that this review can create cyberbullying research programs that can answer these, and other, important basic and applied questions.

## Author Contributions

All authors listed have made a substantial, direct and intellectual contribution to the work, and approved it for publication.

## Conflict of Interest

The authors declare that the research was conducted in the absence of any commercial or financial relationships that could be construed as a potential conflict of interest.

## Publisher's Note

All claims expressed in this article are solely those of the authors and do not necessarily represent those of their affiliated organizations, or those of the publisher, the editors and the reviewers. Any product that may be evaluated in this article, or claim that may be made by its manufacturer, is not guaranteed or endorsed by the publisher.

## Footnotes

^1^We view this “blank slate” approach as essential – if such a sample can be found. Indeed, assessing learning longitudinally would ideally have participants never have engaged in the behavior at baseline and then monitoring changes over time. We are presuming here that youth (vs. adults) would be more likely to have never engaged in cyberbullying or be cybervictimized. However, there may be pockets of the adult population that also have never been involved in these cyber-behaviors, and, therefore, these questions are valid for those adults – if they can be identified.
